# A unique phenotype in a patient with a rare triplication of the 22q11.2 region and new clinical insights of the 22q11.2 microduplication syndrome: a report of two cases

**DOI:** 10.1186/s12887-015-0417-5

**Published:** 2015-08-22

**Authors:** Sara O. Vaz, Renato Pires, Luís M. Pires, Isabel M. Carreira, Rui Anjos, Paula Maciel, Luisa Mota-Vieira

**Affiliations:** Department of Pediatrics of Hospital of Divino Espírito Santo of Ponta Delgada, EPE, Av. D. Manuel I, 9500-370 Ponta Delgada, São Miguel Island, Azores Portugal; Molecular Genetics and Pathology Unit, Hospital of Divino Espírito Santo of Ponta Delgada, EPE, Av. D. Manuel I, 9500-370 Ponta Delgada, São Miguel Island, Azores Portugal; Biosystems & Integrative Sciences Institute (BioISI), Faculty of Sciences, University of Lisboa, 1749-016 Lisboa, Portugal; Cytogenetics and Genomics Laboratory, Faculty of Medicine, University of Coimbra, 3000-354 Coimbra, Portugal; Centro de Investigação em Meio Ambiente, Genética e Oncobiologia (CIMAGO), Faculty of Medicine, University of Coimbra, 3000-354 Coimbra, Portugal; Centre of Neurosciences (CNC), University of Coimbra, 3000-354 Coimbra, Portugal; Department of Pediatric Cardiology, Hospital of Santa Cruz, Av. Prof. Dr. Reinaldo dos Santos, 2790-134 Carnaxide, Portugal; Instituto Gulbenkian de Ciência, Rua da Quinta Grande, 6, 2780-156 Oeiras, Portugal

**Keywords:** 22q11.2 microduplication syndrome, 22q11.2 triplication, Congenital heart defects

## Abstract

**Background:**

The rearrangements of the 22q11.2 chromosomal region, most frequently deletions and duplications, have been known to be responsible for multiple congenital anomaly disorders. These rearrangements are implicated in syndromes that have some phenotypic resemblances. While the 22q11.2 deletion, also known as DiGeorge/Velocardiofacial syndrome, has common features that include cardiac abnormalities, thymic hypoplasia, characteristic face, hypocalcemia, cognitive delay, palatal defects, velopharyngeal insufficiency, and other malformations, the microduplication syndrome is largely undetected. This is mainly because phenotypic appearance is variable, milder, less characteristic and unpredictable. In this paper, we report the clinical evaluation and follow-up of two patients affected by 22q11.2 rearrangements, emphasizing new phenotypic features associated with duplication and triplication of this genomic region.

**Case Presentation:**

Patient 1 is a 24 year-old female with 22q11.2 duplication who has a heart defect (ostium secundum atrial septal defect) and supernumerary teeth (hyperdontia), a feature previously not reported in patients with 22q11.2 microduplication syndrome. Her monozygotic twin sister, who died at the age of one month, had a different heart defect (truncus arteriousus). Patient 2 is a 20 year-old female with a 22q11.2 triplication who had a father with 22q11.2 duplication. In comparison to the first case reported in the literature, she has an aggravated phenotype characterized by heart defects (restrictive VSD and membranous subaortic stenosis), and presented other facial dysmorphisms and urogenital malformations (ovarian cyst). Additionally, she has a hemangioma planum on the right side of her face, a feature of Sturge-Weber syndrome.

**Conclusions:**

In this report, we described hyperdontia as a new feature of 22q11.2 microdeletion syndrome. Moreover, this syndrome was diagnosed in a patient who had a deceased monozygotic twin affected with a different heart defect, which corresponds to a phenotypic discordance never reported in the literature. Case 2 is the second clinical report of 22q11.2 triplication and presents an aggravated phenotype in contrast to the patient previously reported.

## Background

The rearrangements of the 22q11.2 chromosomal region, specifically deletions and duplications, have been known to be responsible for multiple congenital anomaly disorders [1, 2]. The 22q11.2 deletion causes 22q11.2 deletion syndrome, also known as DiGeorge/Velocardiofacial syndrome (DGS/VCFS), which has a prevalence estimated between 1:2000-1:17000 live births [3]. About 90 % of these deletions are *de novo*, while 10 % are inherited from an affected parent [4]. DGS/VCFS is characterized by a wide spectrum of clinical features that include: cardiac abnormalities, particularly conotruncal malformation; thymic and parathyroid hypoplasia; facial dysmorphisms; cognitive delay; palatal defects; velopharyngeal insufficiency; behavioral problems; hearing loss; limb deformity; and feeding difficulty, amongst others. The penetrance of each feature is variable, *i.e.*, no single phenotype happens in all patients and none is obligatory [4–7].

Recently, 22q11.2 microduplication syndrome has been considered a different clinical entity than 22q11.2 deletion syndrome [2]. Recent data suggest that the frequency of duplications is approximately half of the deletions, mainly because duplications are largely undetected − phenotypic appearance is variable, milder, less characteristic, and unpredictable. Inherent methodological issues also contribute to difficulties in the diagnosis of this condition [2, 8–10]. Based on the current literature, the phenotypes of 22q11.2 deletion and 22q11.2 microduplication syndromes sometimes overlap, but this is correlated to ascertainment bias and might represent a slight part of the wide range spectrum of these syndromes [1, 10, 11].

The triplication of the 22q11.2 region was only reported in one study, in which the patient had a very mild phenotype, specifically speech delay, learning difficulties, and dysmorphic features (broad nasal bridge, hand/foot abnormality, epicanthal folds, eversion of the lateral eye lids, and bulbous nasal tip) [10].

In this paper, we report the clinical evaluation and follow-up of two patients affected by 22q11.2 rearrangements that we have recently characterized using molecular techniques [12]. Furthermore, we describe hyperdontia as a new feature of 22q11.2 microdeletion syndrome and report the clinical features of a patient who carries a 22q11.2 triplication, which is extremely important since it is the second case reported in the literature.

## Case presentation

### Case 1

**Fig. 1:**
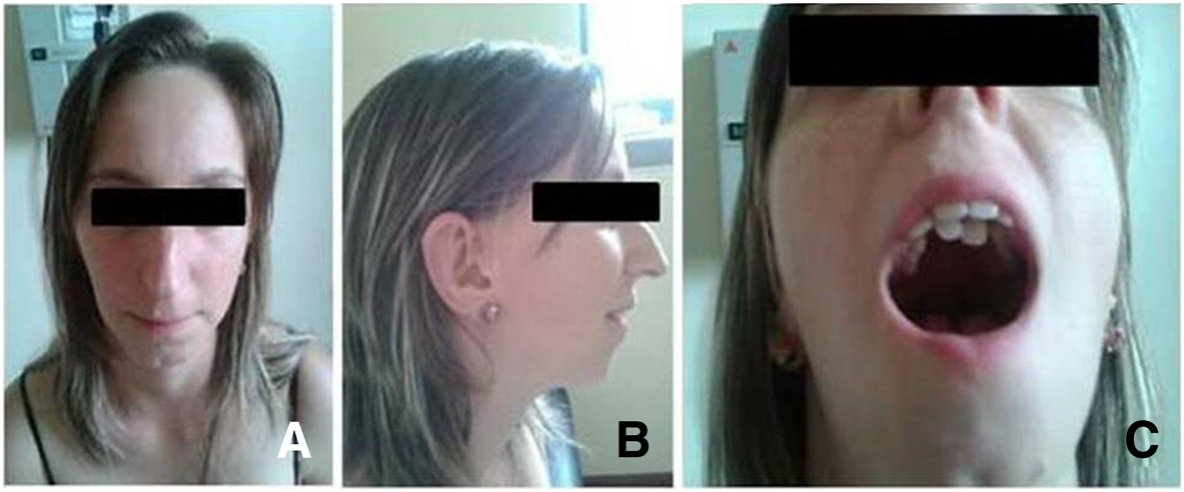
Facial appearance of patient 1 with 22q11.2 microduplication syndrome. **a** Front view. **b** Side view. **c** Hyperdontia

The index case (corresponding to patient C in Pires *et al.* [12]) is a 24 year-old female who is the third child of consanguineous parents (a 26 year-old mother and a 30 year-old father). The patient was born at term (39 weeks of gestation), by vaginal delivery, with a birth weight of 2450 g (5^th^ centile) and length of 47 cm (10^th^ centile). Apgar scores were 1 at the first minute and 10 at the fifth minute. The patient had a monozygotic twin sister affected by truncus arteriosus, who underwent heart surgery, but died at the age of 1 month. The family history revealed another three deceased siblings: the first was a girl who died at the age of 9 months with a possible heart disease; the second was a stillborn boy; the third was a girl who died at the age of 13 months with a diagnosis of pulmonary atresia and ventricular septal defect (VSD). The mother has a history of hyperthyroidism and hypertension; the father has rheumatoid arthritis; both have learning difficulties. Maternal grandparents and the paternal grandfather died of unspecified heart disease. At the age of 9 years, the patient was suspected of having a heart condition due to the presence of a heart murmur and failure to thrive. An echocardiogram showed an ostium secundum atrial septal defect (ASD), which required heart surgery. Facial dysmorphisms were perceived, suggesting DGS/VCFS (Fig. 1a and b). She had recurrent upper respiratory tract infections that required adenoidectomy at the age of 15. She also underwent otorhinolaryngology surgery, due to an infected auricular fistula, and received an otoplasty to correct a pinna deformity (Table 1).

**Table 1:** Clinical characterization of two patients with 22q11.2 alterations and comparison with previously reported cases

Features	Case 1	Other cases^a^	Case 2		Yobb et al., 2005 [10]	
			Patient	Father	Patient	Mother
22q11.2 region	Dup	Dup	Trip	Dup	Trip	Dup
Age at last evaluation (years)	24		20	63	8	
Gender	F		F	M	F	F
Heart defect	+	+	+		−	
Velopharyngeal insufficiency	−	+	+		−	
Palatal defect	+	+	−		−	
Hearing impairment	−	+	+		+^c^	
Failure to thrive	−	+	−		−	
Roncopathy/sleep apnea	−	+	+		−	
Urogenital abnormalities	−	+	+		−	
Cognitive deficits	+	+	+^b^	+	−	
Psychiatric disorders	+	+	+			
Behavioral problems	−	+			+	
Seizures	−	+	−		−	
Headache	+		+^b^			
Hand/foot abnormality	−	+	−		+	+
Dysmorphic features						
− Head						
Microcephaly	−	+	−		−	
Long narrow face	+	+	+			
Proeminent forehead	+	+	−			
− Eyes						
Hypertelorism	+	+	+			
Epicanthal folds	+	+	+		+	
Upslanting palpebral fissures	+	+	−			
Downslanting palpebral fissures	−	+	+			
Superior placement of eyebrows	+	+	−			
Strabismus	−	+	+^b^			
Myopia	+	+	+^b^			
− Nose						
Broad nasal bridge	+	+	−			
Proeminent nose	+	+	+			
Long philtrum	+		+			
Smooth philtrum	+	+	+			
− Ears						
Dysplastic ears	+	+	−			
Preauricular fistula	+	+	−			
− Mouth						
Micrognathia	+	+	−		−	
Retrognathia	+	+	+			
Supernumerary teeth (hyperdontia)	+		−			
Dental cavities	+	+	+			
Additional features						
− Recurrent infections	+	+	+			
− Skeletal						
Hiperlaxity	−	+	−			
Other	−	+	−			
− Dermatologic abnormalities	−		+^b^			
− Allergies	+		+			

^a^According to [2, 10, 23, 24]

^b^Sturge-Weber syndrome’s symptoms that are likely unrelated to the 22q11.2 triplication

^c^Hearing impairment was probably secondary to otitis media

Currently, the patient has mild developmental delay and learning difficulties, hyperdontia (supernumerary teeth; Fig. 1c), and dental cavities. Nonetheless, she is living an independent life and is currently married. In order to investigate the presence of 22q11.2 deletion, which is associated with DGS/VCFS, and other genomic alterations, we performed multiplex ligation-dependent probe amplification (MLPA) and array comparative genomic hybridization (array-CGH), respectively [12]. Both techniques detected a *de novo* duplication (1:2, one normal chromosome and a 22q11.2 duplication in the other) of 2.5 Mb (nucleotide positions 18,894,835 to 21,464,119) [12], supporting the diagnosis of 22q11.2 microduplication syndrome.

### Case 2

This patient, a 20 year-old female who corresponds to patient B in Pires *et al.* [12], is the eighth child of a 42 year-old mother and a 43 year-old father; the latter presents mild intellectual disability and learning difficulties. She was born at 38 weeks of gestation by spontaneous vaginal delivery. The patient has seven normal siblings. At birth, she weighed 2950 g (5^th^ centile), her height was 46 cm (5^th^ centile), and head circumference was 34 cm (25^th^ centile). Apgar scores were 9 at the first minute and 10 at the fifth minute. She had a hemangioma planum on the right side of her face, low set ears, and convergent strabismus on the right eye (Table 1). In infancy, the transfontanelar ultrasound and the computed tomography (CT) scan of the head were both normal. In the first years of her life, she was diagnosed with failure to thrive, developmental delay, hearing impairment, multiple upper respiratory infections, and restrictive VSD and membranous subaortic stenosis, which required surgery. At 13 years of age, a large ovarian cyst was diagnosed.

At 15 years of age, she was diagnosed with Sturge-Weber syndrome. A CT scan showed ocular asymmetry with greater dimensions of the left eye, hypercaption of contrast product in the external posterior wall of the right eyeball, and an almost absent mastoid pneumatization.

Currently, she has moderate cognitive and language impairment, and is dependent for most daily activities. Due to the heart defects, we performed the analysis of the of 22q11.2 region by MLPA, fluorescent *in situ* hybridization (FISH), and array-CGH [12]. MLPA identified four copies of this region, whereas FISH confirmed a tetrasomy (1:3, one normal chromosome and a 22q11.2 triplication in the other) that was most likely inherited from a paternal duplication (1:2) with an extra copy [12]. Moreover, the array-CGH showed a triplication of 3 Mb (nucleotide positions 18,661,724 to 21,917,251) [12].

## Conclusions

The two cases described illustrate the phenotypic variability associated with rearrangements of the 22q11.2 chromosomal region. We establish the similarities and differences between the two patients with the 22q11.2 variants, revealing hyperdontia as a new characteristic of the 22q11.2 microduplication syndrome and new features concerning triplication of the 22q11.2 region.

Only a few patients with 22q11.2 microduplication syndrome and concomitant heart defects have been reported [11, 13]. Patient 1 had an ASD that required surgery. At birth, the diagnosis of DGS/VCFS was suggested due to facial dysmorphisms, failure to thrive, and a strong family history of heart diseases and sudden death syndrome. At that time it was not possible to perform specific techniques to diagnose 22q11.2 duplication. Even though having mild developmental delay, she lives an independent life. It is essential to emphasize two clinical aspects. First, the existence of an anomaly like hyperdontia, which was never reported as being associated to the 22q11.2 microduplication syndrome (Table 1). Second, the fact that her monozygotic twin sister, who died at the age of one month, had a different heart defect, which demonstrate that other factors − post-zygotic genetic, epigenetic, environmental and stochastic − could affect human heart development. These factors may contribute to the discordance of congenital heart disease (CHD) in monozygotic twins [14]. Despite this discordance that has been described in monozygotic twins with DGS/VCFS [15, 16], it was never associated with 22q11.2 microduplication syndrome.

Patient 2, who has a father with 22q11.2 duplication and a normal mother, presents a triplication of the 22q11.2 region and, to our knowledge, this is the second case described in literature [10, 12]. In comparison to the first reported case, who presented speech delay, learning difficulties, and dysmorphic features, patient 2 has an aggravated phenotype characterized by heart defects (restrictive VSD and membranous subaortic stenosis), and presented other facial dysmorphisms and urogenital malformations (ovarian cyst; Table 1). The patient also has a hemangioma planum on the right side of her face. This latter sign is consistent with one of the diagnostic criteria of Sturge-Weber syndrome. As we know, this syndrome is a sporadic congenital neurocutaneous disorder, caused by a somatic activating mutation (c.548G > A, p.Arg183Gln) in *GNAQ* gene [17]. Clinically, it is characterized by the presence of *nevus flammeus* (port-wine stain), involving the area of the first sensory branch of the trigeminal nerve. The second and third trigeminal branches might be involved, by cerebral venous malformations (ipsilateral leptomeningeal angiomatosis) and by glaucoma with ocular capillary venous vascular malformations [18–20]. Sturge-Weber syndrome has been associated with epilepsy, behavioral disorders, cognitive impairment, headaches, spastic hemiparesis, and visual fields defects [18–22]. In fact, patient 2 presents similar features (Table 1) and possibly has the somatic mutation c.548G > A (p.Arg183Gln) in the *GNAQ* gene.

In summary, here we report hyperdontia as a new feature of 22q11.2 microdeletion syndrome. This syndrome was diagnosed in a patient who had a deceased monozygotic twin affected with a different heart defect, which corresponds to a phenotypic discordance never reported in the literature. Moreover, we reinforce that physicians should pay attention to the resemblances between 22q11.2 microduplication and deletion syndromes, in order to avoid misdiagnosis. Therefore, these two syndromes should be considered in the presence of a patient with a dysmorphic face associated with CHD, thymic hypoplasia, developmental delay, and other malformations. Patients with triplication of 22q11.2 region are extremely rare and case 2 presented an aggravated phenotype in contrast to the previously reported, which has a milder phenotype. Both triplication cases fit within the 22q11.2 duplication spectrum. Some factors may contribute to their phenotypic variability, such as differences in genetic background, different genetic dosages, abnormalities in the parent of origin or imprinted chromosomal material (*e.g.*, uniparental disomy). Finally, we suggest that duplications and triplications could be underdiagnosed, particularly if evaluated by FISH in metaphase spreads. Molecular tests, like MLPA or array-CGH, could be more adequate.

### Consent

This work was approved by the Health Ethics Committee from the Hospital of Divino Espírito Santo of Ponta Delgada, EPE. Written informed consent for the patients’ inclusion in the study was obtained from themselves and their parents. Patient 1 gave informed consent to publish her clinical information and photos. Patient 2 authorized the publication of her clinical data, however she did not allow physicians to publish her photos. The copies of the written consents are available for review by the Editor of this Journal.

## Abbreviations

DGS/VCFS: DiGeorge/Velocardiofacial syndrome
VSD: Ventricular septal defect
ASD: Atrial septal defect
MLPA: Multiplex ligation-dependent probe amplification
array-CGH: Array Comparative genomic hybridization
CT: Computed tomography
FISH: Fluorescent *in situ* hybridization
CHD: Congenital heart disease
